# A Dataset of Metaphors from the Italian Literature: Exploring Psycholinguistic Variables and the Role of Context

**DOI:** 10.1371/journal.pone.0105634

**Published:** 2014-09-22

**Authors:** Valentina Bambini, Donatella Resta, Mirko Grimaldi

**Affiliations:** 1 Center for Neurocognition and Theoretical Syntax, Institute for Advanced Study (IUSS), Pavia, Italy; 2 Centro di Ricerca Interdisciplinare sul Linguaggio (CRIL), University of Salento, Lecce, Italy; University of Rome, Italy

## Abstract

Defining the specific role of the factors that affect metaphor processing is a fundamental step for fully understanding figurative language comprehension, either in discourse and conversation or in reading poems and novels. This study extends the currently available materials on everyday metaphorical expressions by providing the first dataset of metaphors extracted from literary texts and scored for the major psycholinguistic variables, considering also the effect of context. A set of 115 Italian literary metaphors presented in isolation (Experiment 1) and a subset of 65 literary metaphors embedded in their original texts (Experiment 2) were rated on several dimensions (word and phrase frequency, readability, cloze probability, familiarity, concreteness, difficulty and meaningfulness). Overall, literary metaphors scored around medium-low values on all dimensions in both experiments. Collected data were subjected to correlation analysis, which showed the presence of a strong cluster of variables—mainly familiarity, difficulty, and meaningfulness—when literary metaphor were presented in isolation. A weaker cluster was observed when literary metaphors were presented in the original contexts, with familiarity no longer correlating with meaningfulness. Context manipulation influenced familiarity, concreteness and difficulty ratings, which were lower in context than out of context, while meaningfulness increased. Throughout the different dimensions, the literary context seems to promote a global interpretative activity that enhances the open-endedness of the metaphor as a semantic structure constantly open to all possible interpretations intended by the author and driven by the text. This dataset will be useful for the design of future experimental studies both on literary metaphor and on the role of context in figurative meaning, combining ecological validity and aesthetic aspects of language.

## Introduction

We produce approximately one novel metaphor every 25 words, based on an estimation on TV programs [1]. This is one of the many hints suggesting that metaphor, as a paradigmatic case of non-literalness, is a pervasive phenomenon in human communication and cognition, possibly a hallmark of conceptual and linguistic abilities exclusive of human communication [2]–[4]. In the last thirty years, metaphor has become a topic of investigation for cognitive linguistics and pragmatics, and much work in psycholinguistics and cognitive neuroscience has examined the features and mechanisms of metaphor comprehension, by employing a variety of paradigms and carefully built materials simulating the metaphorical expressions used in everyday conversation. “My lawyer is a shark” [5] is just one above all typical stimuli, which usually come on a par with equivalent literal controls.

A long-lasting debate in the experimental literature concerns whether metaphor is a departure from a “literal norm” [6], and thus accessed indirectly after the rejection of the literal meaning, or rather it is understood as quickly and automatically as literal utterances [5]. Longer reading times to metaphorical than to literal utterances were taken as an index of the access to a default literal meaning. However, equally strong evidence showed that, when utterances are embedded in richer context, the difference between metaphorical and literal utterances is much reduced in terms of reading times [7]. This pattern was taken as evidence for the “direct access” [8], which argues that processing metaphors involves a single mechanism that is sensitive both to linguistic and non-linguistic information, and that lexical and contextual levels interact in the very early stages of metaphor comprehension. The Graded Salience Hypothesis [9] provides an alternative suggestion to reconcile the direct and indirect view by overcoming the distinction between literal and metaphorical language and introducing the distinction between salient and non-salient meanings. The access to both literal and metaphorical meaning is considered salience-sensitive, thus salient meanings —i.e., familiar, frequent, conventional, and prototypical—are accessed directly regardless of context and emerge even when contextually inappropriate [9]. Recent ERP evidence suggests that metaphor comprehension unfolds in different phases linked to the N400 and the P600 effects [10], and that literal meaning aspects might indeed be activated in the earlier stages of processing [11], supporting “the lingering of the literal” view [12]. Overall, experimental pragmatics has shown that greater efforts for metaphors as compared to literal expressions might index additional lexical-pragmatic adjustments operating at the conceptual level [13], as well as extra cognitive benefits [14]. Neuropragmatics too has devoted attention to metaphor processing, showing the involvement of a bilateral pattern of activations, related to linguistic as well as non-linguistic processes, especially mindreading [15]–[17].

Yet metaphor is better known in our encyclopedic knowledge for much more aesthetically relevant examples than the lawyer-shark case, for instance the Shakespearian “All world’s a stage” (from *As you like it*) or “blanket of the dark” (from *Macbeth*). Besides being a frequent phenomenon in everyday communication, metaphor is indeed a powerful tool in the poet’s armamentarium, generating evocative images that hit the readers’ minds. 11% of the lexical units in fiction are metaphor related [18], and one can assume a similar or even higher percentage for poems and other literary genres. Metaphor as a literary tool has been a topic of investigation in rhetoric since old times [19], with emphasis on metaphor as an important mark of style [20]. Modern studies have opened to considerations coming from cognitive and pragmatic approaches, with a great deal of cross-fertilization [21]–[23]. On the pragmatic, relevance-theory inspired side, literary metaphor is described in terms of additional cognitive efforts in the interpretation process, characterized in terms of open-endedness and generation of a wide array of weakly communicated implicatures that contribute to form a poetic halo [24]. On the cognitive poetics side, literary metaphor is characterized by the logical contradictions between the two terms that are mapped onto one another in the metaphorical expression, resulting in specific and unique effects [25]. Other authors emphasized that metaphors are generally visible in a text as an “overt incongruity”, that is an apparently anomalous expression validated according to a sort of intratextual norm [26], and combine with the structure of the text generating “ministories of disnarrated materials” [27], pointing in the direction of a narratological approach to figurative language [23].

A challenging topic in metaphor studies concerns the relationship between everyday metaphors and literary metaphors. Old debate marked the distinction between the continuity and the discontinuity view, debating whether literary metaphor and everyday metaphor are supported by the same or different mechanisms. Nowadays most scholars argue that both positions are correct in elucidating different aspects of figurative expressions in literature. Most also claim that advances in the field can be achieved only by incorporating empirical investigations, possibly comparing everyday and literary metaphors through corpus-linguistic and psycholinguistic research [28]. Initial evidence in this direction comes from behavioral studies comparing subjects’ judgments. Literary metaphors are rated as more difficult than journalistic metaphors [29], and as more meaningful than randomly constructed comparisons [30]. Interestingly, automatically generated anomalous comparisons are slower to be rejected when subjects suppose they were written by poets, showing that the identification of the authorial intention, i.e., the author’s communicative and aesthetic goals in making the expression, plays an important role when it comes to interpreting literary materials [31]. Poetic metaphors have also been included in sets of novel metaphors and compared to conventional metaphors, which led to the description of effects related to novelty, although not specific to literary materials [32], [33]. Apart from this fragmented evidence, up to now experimental investigations have been mostly concerned with everyday metaphor, and the specific features of literary metaphors remain without empirical support.

In this article we attempt to pave the way for an “experimental turn” in the study of literary metaphor by providing a dataset of materials extracted from original texts, based on considerations coming from the extensive experimental literature on everyday metaphor. This approach goes in the direction of the current requirements of empirical investigation on language, namely the need of controlled materials and ecological validity, two aspects that deserve some considerations.

In the last years the need for datasets suitable for experimental studies has increased. In order to achieve the maximum experimental control, stimuli often need to be selected according to specific attributes that modify behavioral and neural responses, and ready-made datasets of norms and ratings that satisfy the preferred constraints are of great benefit [34]. This need has recently emerged also for metaphor. Both psycholinguistic and neurolinguistic studies have indeed devoted a great effort to define the factors that influence metaphor comprehension. Special attention has been paid to the role of conventionality, suggesting a progressive shift from comparison to categorization as metaphors become conventionalized [35], familiarity, suggesting different inferential chains for familiar and unfamiliar figurative expressions [36], and meaningfulness and difficulty, to ensure interpretability [13]. Also the neural response to metaphor is significantly affected by different degrees along these variables [37], [38]. To facilitate research, datasets that include norms and ratings for these variables are being created for different languages. For English, norms are available for everyday metaphors along the dimensions of familiarity, naturalness, imageability, concreteness, figurativeness and interpretability [39]. For Italian, datasets of different conventionality are available [13], [40]. For literary metaphor, Katz and collegues [41] pioneered the field by collecting scores along a vast array of variables (comprehensibility, ease of interpretation, degree of metaphoricity, metaphor goodness, metaphor imagery, subject/tenor imagery, predicate/vehicle imagery, felt familiarity, semantic relatedness, and number of alternative interpretations) on a corpus of metaphors extracted from English literary works and rewritten, when necessary, to conform to the form “A is B”. However, this remains an isolated study, with the limitation of including not fully original materials, not further explored and not extended to other languages.

On a side with the need of controlled stimuli, research seems to go in the direction of increasing the ecological validity of the experimental paradigms, especially when it comes to pragmatics and context [42]–[44]. As shown above, most scholars believe that context supports metaphor comprehension and reduces the difference between figurative and literal meanings [45], [46]. Despite this, the majority of the studies consider isolated metaphors, either word pairs or single sentences, and only occasionally experimental materials include context, mostly in the form of few supportive words [15] or short stories of a paragraph length [47]. In the case of literary metaphor, the context of poems and novels is likely to be key to figurative comprehension, in a way that might differ from the context of everyday discourse. Indeed, in natural reading of poems and novels, the interpretative activity cannot be confined to the figurative expression but implicates larger chunks of context [26]. This domain, however, remains unexplored, as well as the role of context with respect to other psycholinguistic variables.

The goal of our study was twofold. First, we aimed to provide the first dataset of original Italian literary metaphors described along the major psycholinguistic variables. Second, we aimed to extend the study to include the condition when natural reading of literary metaphors in context is preserved. This could also allow to explore the materials in terms of interplay of variables that affect the comprehension process in and out of context, possibly contributing to shed initial light on literary metaphor comprehension and appreciation. We collected metaphorical phrases of the form “A of B” from original texts of representative Nineteenth and Twentieth century Italian authors, e.g., *prato di velluto*/*grass of velvet* (Gozzano, *The Youthful Error*). In Experiment 1, phrases were tested in isolation (out of context) in order to get information on the lexical-pragmatic processes in interpreting the expressions, i.e., how literal word meanings are modified to make sense of the phrases, even in the absence of wider interpretational cues. In Experiment 2, phrases were embedded in context, i.e., the original texts, in order to assess how judgments vary in ecologically valid literary contexts. Based on the previous literature, we selected a number of psycholinguistic variables that are standard in empirical approach to metaphor and well known for influencing the processing of everyday metaphor, and that might presumably influence literary metaphor as well. The dataset of literary metaphors, along with the original literary contexts and the scores described in the two Experiments that follow, is available for downloading from File S1. Literal (word by word) translations into English are also provided.

### Rationale for Selected Psycholinguistic Variables

The major variables selected from the experimental literature and used here to score the dataset are listed below, including, when possible, predictions on the expected results in and out of context. Predictions are mostly based on evidence collected for everyday metaphor and discourse context. However, the literary nature of the materials may yield unexpected findings, reflecting the features identified by pragmatics and cognitive poetics as specific to literary metaphor, among which the open-endedness of the interpretative process [24] and its relation with the textual structure [26]. Three variables, namely word frequency, phrase frequency and readability (the latter only in context), were measured by using automatic methodologies. Five variables, namely cloze probability, familiarity, concreteness, difficulty, and meaningfulness were assessed through behavioral tasks. Most of the variables were applied at the level of the metaphorical phrase (cloze probability, familiarity, concreteness, difficulty, meaningfulness), while frequency was measured both for phrases and single words, and readability refers to texts.

#### Frequency

Frequency is a property that refers to the extent to which a specific word is encountered in a particular language either in written or oral form. It plays an important role in several cognitive processes including word processing and accessibility of lexical representations in memory [48]. In metaphor studies, frequency at the word-level is usually assessed through databases [39], and recently also at the phrase-level through the web [49]. In our study, values of word frequency were extracted from a corpus and frequency dictionary of written Italian (CoLFIS, [50]) accessed through the *EsploraColfis* web-interface [51] for the first and the second content word of the phrase, while phrase frequency was calculated in the Google search engine. As literary metaphors were extracted from modern sources not containing archaic and obsolete terms, word frequency range is expected to report medium-high values, similarly to word frequency in everyday metaphors (e.g., [13], [15]). Phrase frequency is likely to report low values due to the literary origin of the materials.

#### Readability

When texts are included as experimental materials, readability becomes an important parameter, being based on objective features of texts and traditionally associated with overall text quality [52]. Here readability was measured through the Gulpease index [53], an index developed for the Italian language similar to the Flesch’s Reading Ease used for the English language [54], which was applied to the text excerpts containing the metaphorical phrases. The Gulpease index considers two linguistic variables, namely word length (mean number of letters) and sentence length (mean number of words per sentence), and returns a value indicating the ease of reading for populations with different degrees of formal education. Although the excerpts vary in syntactical structure, we expect the readability values to be homogeneous, due to the common literary origin, and to fall into the range indicating easy comprehension for the undergraduate population. Even if useful for a general objective evaluation of the text, values from this computation should be considered only partially reliable. Drawbacks are principally linked to the lack of difference between genres and, more importantly, to the impossibility of measuring the presence of figurative language, which may influence comprehension even when the readability index is constant [55].

#### Cloze probability

Cloze probability is the probability that a given word will be produced in a given context on a sentence completion task [56], with major effects on word recognition and integration in context [57]. In metaphor studies, cloze probability is usually held at very low levels and constant across experimental conditions to minimize nuisance effects [15], [58]. Here we measured cloze probability of the literary metaphors both in the absence and in the presence of context, thus assessing also potential differences due to the amount of textual information. Previous evidence showed that cloze probability is very low for everyday metaphors [58], but becomes higher when context is provided [15]. Literary metaphors are likely to be unpredictable in both cases, possibly with higher values in the contextualized condition, also reflecting previous exposure to the materials, maybe in schooling. Yet metaphors come as unexpected incongruities in the literary texts, and this might have repercussions on cloze probability.

#### Familiarity

Familiarity reflects how often a subject has been exposed to a particular statement either in written or oral form [59]. It does not overlap with frequency, as an item may be frequent on a lexical database but unfamiliar to a single individual [9]. Familiarity is thus best defined as frequency of experience or “felt familiarity” [41]. This variable proved to influence both visual and auditory word recognition in a number of tasks [60]. It is also frequently addressed in metaphor studies [13], [38], [41], [61] because, contrary to frequency, it is able to provide information about metaphorical meaning as perceived by language users. Unfamiliar metaphors are often named “novel” [37] or “unconventional” [38], which leads to an overlap of familiarity, novelty, and conventionality concepts. However, while novelty and familiarity seem to refer to the two extremes of the same scale, conventionality seems to indicate something different. In particular, familiarity reflects repeated exposure to a given combination of terms in metaphorical statements, while conventionality reflects repeated figurative use of a specific term [62], pointing in the direction of lexicalization and idiomaticization. In this study, we focus on the familiar/unfamiliar distinction, assuming that literary metaphors are non-conventional and non-lexicalized by definition. Due to their originality, literary metaphors are likely to be judged as unfamiliar when presented in isolation. When presented in context, we might expect that textual information facilitates overall comprehension and increases familiarity, as it happens for everyday metaphor [9]. It should be noted, however, that the context of literary texts is different from the context of everyday conversation, and it might introduce elements that enhance the interpretative activity and make the figurative expression more open to different interpretations, thus not increasing familiarity, even when subjects might have been previously exposed to those materials [26].

#### Concreteness

The concept of concreteness operationalizes the distinction between what exists in the physical world and what exists in the human mind [63]. Concrete words are claimed to be more grounded in the perceptual experience and more quickly and easily processed than abstract words [64]. Cognitive linguistics approaches to metaphor emphasized that highly concrete expressions are frequently used to explain less concrete concepts [2], and most metaphors in literature (about 51%) consist indeed of two concrete nouns [65]. Experimental studies are beginning to include concreteness as one of the factors affecting metaphor comprehension. Cardillo and colleagues [39] derived concreteness values both for each of the words forming the metaphors and for the metaphorical sentences, by averaging the values associated with all content words. In our study, metaphors of the form “A of B” were treated as single meaning units, in order to get information of the concreteness of the interpretation of the expression. Following previous rating studies on the concrete-abstract distinction beyond the word level [66], subjects were asked to rate the concreteness of the whole metaphorical phrase, both in isolation and in context. Since our metaphors were mostly identified in the corpus starting from concrete nouns (e.g., types of physical location and types of physical events) and tended to have a concrete-concrete or concrete-abstract structure (see section “Stimuli” below), we expect medium-high concreteness scores both in and out of context. Yet, as discussed for familiarity, literary context might introduce a setting that fosters multiple interpretations, possibly including the generation of abstract meanings and thus reducing the concreteness values of the expression.

#### Difficulty

Difficulty measures the effort required for achieving successful comprehension, thus providing a subjective measure of perceived ease of comprehension. Difficulty of metaphor interpretation is frequently assessed in behavioral as well as ERP and imaging studies [13], [39], [67], [68], as stimuli that are judged as more difficult might be processed differently, for instance in terms of hemispheric recruitment [38]. Being novel and literary, our metaphors will probably be perceived as difficult to understand, also in light of the density of the “A of B” structure. Context, however, might reduce the difficulty of the process.

#### Meaningfulness

Meaningfulness is usually defined as the subjects’ confidence in understanding what an expression actually means [69]. It is an important dimension of metaphor, as it reflects the interpretability of the expression, one feature that is known to be sensitive to the role of authorial intention [31], and that becomes crucial when metaphor need to be distinguishable from anomalous expressions included in the experimental materials [13]. Meaningfulness is usually measured in behavioral as well as ERP and imaging studies on metaphors, either in pre-tests as an index of interpretability [70], or during the test for receiving a feedback on the materials and enhancing comprehension [37]. Here we collected meaningfulness scores in order to assess whether literary metaphorical phrases out of context are recognized as meaningful and whether literary context facilitates the construction of a meaning. Subjects were not told about the literary origin of the material in the out of context condition, while they were informed in the contextualized presentation, in order to avoid judgments simply based on a superficial and clearly visible difference between poetic texts and newspaper extracts (used as control condition).

Furthermore, based on previous evidence on the relations between psycholinguistic variables [41], [61], we expected that for literary metaphors the different dimensions constitute a cluster that go in the same direction without being completely overlapped. Specifically, we expect a strong correlation between familiarity and difficulty [41], and between familiarity and difficulty [41], [61]. As for phrase frequency, we expected correlation with meaningfulness and familiarity, as generally reported in the literature not specific to metaphor [71]. Furthermore, we acknowledged that context could alter these trends in terms of both mean values and correlations among the variables. Assuming that literary context behaves like everyday discourse context, we expected it to increases cloze probability, familiarity, concreteness and meaningfulness, while reducing difficulty.

## Experiment 1: Literary Metaphors Out of Context

### Method

#### Ethics Statement

This study is part of a wider research aiming at investigating the neural correlates of literary metaphor through behavioral and ERP data. We obtained the authorization of the Ethical Committee of Lecce ASL for the whole protocol. While informed consent was requested for the ERP study, it was waived by the ASL/LE Committee for the behavioral part of the study described here, given that it involved simple ratings on linguistic expressions. All participants agreed to participate after receiving detailed explanations of the procedure of the questionnaires. All data were treated anonymously.

#### Participants

One hundred-five students (83 F, mean age  = 23, SD  = 4.31) of the Foreign Language Faculty (University of Salento) volunteered in Experiment 1. All participants were Italian native speakers. In order to obtain unbiased ratings, subjects were unaware of the literary origin of the metaphors.

#### Stimuli

The stimuli were literary metaphors extracted from Italian poems and novels of the most representative Nineteenth and Twentieth century authors, among which D’Annunzio, Pirandello, Pascoli, Eco, Ungaretti, Montale, Campana. A sample of texts by each author was collected and uploaded in the open source AntConc 3.2.1 software for corpus exploration. Starting from keywords belonging to semantic classes that are considered particularly productive sources of metaphor (i.e., types of physical location, e.g., sea, river, sky, etc., or types of physical events, e.g., rain, sunset, storm, etc.) [72], the corpus was searched for metaphorical phrases of the form “A of B” (e.g., *prato di velluto*/*grass of velvet*). All phrases were read over by three linguists independently, who removed potentially problematic items and judged the expressions as metaphorical. The final set comprised 115 literary metaphors, among which 58 were from poems and 57 from novels.

Genitive metaphors are especially frequent in literary texts, as they seem to open the way to multiple interpretations. This choice allowed for the selection of a sufficiently high number of homogeneous stimuli, avoiding any type of rewriting as done in previous norms on literary metaphor. Although the genitive structure may be syntactically different from the more commonly tested structure “A is B”, the process of meaning construction is likely to be on a par with the analogy between copular construction and complex noun phrases of the type “this kind of flower” [73], [74]. In terms of surface metaphor structure, some expressions displayed the standard tenor-vehicle order (e.g., *folla di pietra*/*crowd of stone*), some the opposite order (e.g., *finestra dell’anima/window of the soul*). In terms of mapping, directionality had concrete-concrete or concrete-abstract structure, since most of the keywords used for searching the corpus were represented by concrete nouns.

The stimulus set also included control phrases, namely literal phrases and semantically anomalous phrases, to help subjects anchor responses along the scale. In order to generate these control conditions, the last word of the metaphorical phrase (“smoke” in the previous example) was held constant, while the first word was replaced with a word of similar frequency and length (in number of letters) in order to create literal phrases (e.g., *divieti di fumo*/*prohibitions of smoke*)—attested in Italian newspapers—and anomalous, nonsense phrases (e.g., *chicchi di fumo/grains of smoke*). Here only data on literary metaphor are analyzed. Examples are reported in Table 1; see Table S1 in File S1 for the complete set of literary metaphors.

**Table 1:** Examples of literary metaphors in isolation (Experiment 1) and in original contexts (Experiment 2).

Author and source	Experiment 1	Experiment 2
G. Gozzano, “Il Giovenile Errore”	**prato di velluto**	In questo brano un uomo si interroga sulla sua esperienza. “Non so se veramente fu vissuto/Quel giorno della prima primavera./Ricordo o sogno? Un **prato di velluto**,/Ricordo o sogno? Un cielo che s’annera.”
*G. Gozzano, “The Youthful Error”*	***grass of velvet***	*In this passage a man is wandering about his experience. “I don’t know if really was lived*/*that day of the early spring*/*Do I remember or dream? A * ***grass of velvet*** *,*/*Do I remember or dream? A sky that grows dark.”*
G. Pascoli, “Myricae”	**cielo di perla**	In questo brano si descrive un paesaggio naturale con animali e piante. “Tra gli argini su cui mucche tranquilla-/mente pascono, bruna si difila/la via ferrata che lontano brilla;/e nel **cielo di perla** dritti, uguali,/con loro trama delle aeree fila/digradano in fuggente ordine i pali.”
*G. Pascoli, “Myricae”*	***sky of pearl***	*In this passage a natural landscape with animals and plants is described. “Between embankments, where the cattle graze*/*in peace, the railroad stretches out in a straight,*/*dark brown line that glimmers from afar;*/*in the * ***sky of pearl*** *, the telegraph poles create*/*another line in their aerial plot beside*/*the tracks, and in shrinking order, disappear.”*

Original Italian, English translation in italics (for Myricae, the English version is adapted from the translation by Ryan Snyder). In Experiments 2, the original context is preceded by an introductory sentence.

#### Procedure

Word frequency was measured on the CoLFIS corpus [50]. This corpus comprises over three million words from contemporary written Italian texts reflecting the reading habits of the Italian population. The corpus can also be explored through a web interface, allowing for list search [51] Phrase frequency was calculated through the Google search engine (updated to February 2012).

In the cloze probability task, literary metaphors and control phrases were divided into three lists using a Latin-Square design and in pseudo-randomized order. Each list was administered to a different group of 15 subjects. Phrases were truncated after the prepositions (e.g., “somersaults of …”) and subjects were asked to complete the fragments with the first word that came to mind.

In familiarity, concreteness, difficulty, and meaningfulness tasks, two randomized lists for each variable were created and each subject rated the phrases in two of the three possible experimental conditions (metaphorical, literal, anomalous). Each randomized list was administered to a different group of 15 participants. Each participant received an experimental booklet, including one page of instructions with a few examples and a randomized list of items, and was asked to rate each item on two scales, either concreteness and familiarity, or difficulty and meaningfulness. Each item was assessed by 15 raters on 5-point scales (1 =  *very unfamiliar/abstract/very easy/meaningless*; 5 =  *very familiar/very concrete/very difficult/very meaningful*). Participants were tested in groups in a classroom and the ratings were untimed. Each session lasted approximately 45 minutes.

### Results

#### Descriptive statistics

Summary statistics for objectively measured variables (lexical frequency and phrase frequency) and behaviorally assessed variables (cloze probability, familiarity, concreteness, difficulty, and meaningfulness) are shown in Table 2.

**Table 2:** Descriptive statistics of Experiment 1 (out of context presentation).

Variable	Min	Max	M	SD	Mdn	α	Skew	95% CI
First word frequency^a^	0.30	2.99	1.72	0.58			−0.35	0.11
Second word frequency^a^	0.30	2.99	1.96	0.62			−0.40	0.11
Phrase frequency^a^	0.30	6.47	3.93	1.89			−0.82	0.35
Cloze probability	0	0.27	0.01	0.04	0		4.91	0.01
Familiarity	1.27	3.87	2.24	1.27	2	0.78	0.78	0.06
Concreteness	1.13	3.53	2.07	1.17	2	0.78	0.89	0.05
Difficulty	1.93	4.07	3.03	1.16	3	0.76	0.01	0.05
Meaningfulness	1.60	3.93	2.63	1.13	2	0.79	0.48	0.05

Frequency (first word, second word, and phrase), Cloze probability, Familiarity, Concreteness, Difficulty, and Meaningfulness of 115 literary metaphors out of context. Minimum and maximum values, means, standard deviations, medians, α values, skewness values, and 95% confidence intervals are provided for each variable.

a Values were log_10_ transformed.

CoLFIS word frequency was log-transformed to better approximate a normal distribution [48]. Both words reported medium-high frequency on average. For the first word of the phrases, the average value was 1.72 and ranged from less frequent words like *capriole/somersaults* (0.30) to more frequent words like *occhi/eyes* (2.99). For the second word of the phrase, the average frequency was 1.92 and ranged from *tenebre/darkness* (0.30) to, again, *occhi/eyes* (2.99). Overall, the first nouns of the phrases had lower frequency than the second nouns [t (228) = −3.047, p<0.05].

Phrase frequency measured in terms of Google-generated frequency counts was also log-transformed. Average frequency of the whole set of phrases was quite low (M = 3.93). Values ranged from *alito di sepolcro/breath of grave*, *scintilla di senape/spark of mustard*, *nidi d’illusione/nests of illusion*, and *brivido di ferro/shiver of iron*, which were the least frequent (log-transformed value 0.30), to *goccia di luce/drop of light*, which was the most frequent (log-transformed value 6.47).

Cloze probability was calculated by dividing the number of subjects who completed phrase fragments with the target word (i.e., the word occurring in the original phrase) by the total number of tested subjects (i.e., N = 15). Words similar to the original or synonyms were not counted as correct responses [75]. Cloze probability of literary metaphors was very low, 1% on average. Most phrases were totally unpredictable (cloze probability equal to 0%). The most predictable phrases proved *finestra dell’anima/window of the soul* (M = 27%) and *velo d’ombra/veil of shadow* (M = 20%).

For behaviorally assessed variables, a good discriminatory validity emerged from the use of all scale points. Cronbach’s alpha was 0.79 for meaningfulness ratings, 0.78 for familiarity and concreteness ratings, 0.76 for difficulty ratings (N = 15). These agreement levels suggested that the average ratings across participants could be used for further analyses on the relationship between the variables.

Familiarity judgments of the overall set scored around low values (M = 2.24; Mdn = 2; 95% CI [2.18, 2.3]). Values ranged from *piede di vento/foot of wind*, which was considered the least familiar (M = 1.27; Mdn = 1; 95% CI [1.02, 1.52]), to *ombra di tristezza/shadow of sadness* and *onda di tristezza*/*wave of sadness*, which were considered the most familiar (M = 3.87; Mdn = 4; 95% CI [3.15, 4.59]). On the concreteness scale, the set of literary metaphors reported low values on average (M = 2.07; Mdn = 2; 95% CI [2.01, 2.12]). In particular, scores ranged from *semi di dolore*/*seeds of sorrow* (M = 1.13; Mdn = 1; 95% CI [0.93, 1.32]), which proved the least concrete item, to *fili di pioggia/threads of rain* that was the most concrete of the list (M = 3.53; Mdn = 3; 95% CI [2.94, 4.12]). Overall difficulty scores were distributed around the central value of the scale (M = 3.03; Mdn = 3; 95% CI [2.97, 3.08]). The lowest value was reported for *abbraccio del sonno/embrace of the sleep* (M = 1.93; Mdn = 2; 95% CI [1.54, 2.32]), and the highest for *zuffa di clamori*/*brawl of clamors* (M = 4.07; Mdn = 4; 95% CI [3.54, 4.60]). Finally, literary metaphor reported overall medium-low meaningfulness scores (M = 2.63; Mdn = 2; 95% CI [2.56–2.68]), with a range that extended from *fiore della fiamma*/*flower of the flame* (M = 1.60; Mdn = 2; 95% CI [1.25, 1.95]) to *canto delle foglie/song of the leaves* (M = 3.93; Mdn = 4; 95% CI [3.44, 4.42]).

#### Relations among variables

To assess the relations among variables, Spearman’s rank-order coefficients (r_s_) were calculated. The matrix is shown in Table 3. Since all variables were measured on the phrase with the exception of word frequency, this last variable was not included in the correlation matrix. Google log-transformed phrase frequency correlated positively with meaningfulness (r_s_ (113) = 0.44, p<0.01) and familiarity (r_s_ (113) = 0.31, p<0.01), and negatively with difficulty (r_s_ (113) = −0.40, p<0.01). Thus, more frequent phrases were rated as more meaningful, more familiar and less difficult. However, correlation values were not very high and accounted only for the 15% of variance on average.

**Table 3:** Spearman’s correlation coefficients in Experiment 1 (out of context presentation).

Variable	1	2	3	4	5	6
**1. Phrase frequency** a	–	0.09	0.31**	0.01	−0.40**	0.44**
**2. Cloze Probability**		–	0.16	−0.07	−0.15	0.23*
**3. Familiarity**			–	0.46**	−0.60**	0.69**
**4. Concreteness**				–	−0.19*	0.17
**5. Difficulty**					–	−0.88**
**6. Meaningfulness**						–

Familiarity- concreteness and difficulty-meaningfulness were within subjects correlations.

a Values were log_10_ transformed.

* p<0.05; ** p<0.01.

As concerns variables assessed behaviorally, the strongest correlation was reported between difficulty and meaningfulness (r_s_ (113) = −0.88, p<0.01), capturing the 78% of variance. More difficult items were also rated as less meaningful. A second strong correlation was reported between meaningfulness and familiarity (r_s_ (113) = −0.69, p<0.01), accounting for the 48% of variance. This suggested that more meaningful metaphors were perceived as more familiar as well. An inverse robust correlation between difficulty and familiarity (r_s_ (113) = −0.60, p<0.01), explaining the 36% of variance, and a positive correlation between concreteness and familiarity (r_s_ (113) = −0.45, p<0.01), capturing the 21% of variance, were observed. In sum, both less difficult and more concrete items were felt as more familiar. Finally, weak correlations were reported between cloze probability and meaningfulness (r_s_ (113) = 0.23, p<0.05) showing that more predictable items were also rated as more meaningful, and between concreteness and difficulty (r_s_ (113) = −0.19, p<0.05) showing that more concrete items were rated as less difficult to understand. However, both correlations were not highly reliable because they explained only the 4% and 5% of variance, respectively.

### Interim discussion

Overall, the data obtained for the out of context presentation showed that (a) literary metaphors were judged as unpredictable, not very familiar, abstract, moderately difficult to understand yet meaningful; (b) automatically measured phrase frequency interacted with the rated psycholinguistic variables, in particular with meaningfulness; (c) a cluster of variables with internal consistency emerged; (d) the variables correlated with one another and the strongest correlations were recorded among three variables, namely meaningfulness, difficulty, and familiarity. Although the data were obtained from a limited number of participants, the narrow confidence intervals seem to confirm the precision of our estimates and the Cronbach’s alpha confirmed the reliability of the mean ratings.

Overall, the data scored around medium-low values on all dimensions, similarly to English literary metaphor [41] and in line with novel everyday metaphor [39]. The major consideration stemming from these findings, and specifically from the correlations, is the need to consider multiple attributes while assessing literary metaphor. Again, this is consistent with previous results for English literary metaphors [41], [76], showing a strong correlation among several dimensions and suggesting the presence of an underlying “monster factor” that governs literary metaphor comprehension. Specifically, we replicated the strong correlation between familiarity and difficulty observed in Katz et al.’s study [41]. A similar pattern is visible also for everyday predicate and nominal metaphors, with variables like familiarity, naturalness, imageability, figurativeness, and interpretability strongly correlated [39]. We also observed correlations between phrase frequency and meaningfulness, and between phrase frequency and familiarity. This resembles previous findings on non-metaphorical materials [77] and does not seem to be a specific feature of metaphor. However, the absence of a proper context might have influenced the interpretative process in a way that alters the natural conditions of reading literary texts and affects the rating on the different variables, for example increasing difficulty and lowering familiarity and meaningfulness scores. Experiment 2—where literary metaphors were embedded in their original contexts—was aimed at clarifying these issues.

## Experiment 2: Literary Metaphors in Context

### Method

#### Participants

One hundred-eighty students (145 F, mean age  = 20, SD = 2.5) of the Foreign Language Faculty (University of Salento) that did not participate in Experiment 1 volunteered in Experiment 2. Ethical procedures were as in Experiment 1.

#### Stimuli

The items of Experiment 2 were a selection of the items of Experiment 1, provided with the original contexts. Only literary metaphors embedded in text excerpts that were sufficiently coherent for interpretation were included. The selection was based on three independent judgers and returned 65 items, among which 32 were poem excerpts and 33 novel excerpts. Text length was 50 words on average. Examples are provided in Table 1; see Table S2 in File S1 for the complete set, including the original contexts.

Corresponding literal phrases—selected as in Experiment 1—embedded in texts extracted from Italian newspapers and magazines were included. Anomalous expressions were not used for this experiment. For both literary metaphors and literal phrases, texts were preceded by an introductory sentence to prevent subjects from additional processes of search for coherence, as it happens for the presentation of untitled texts [78]. Only data concerning literary metaphor are analyzed and discussed here.

#### Procedure

The readability Gulpease index was assessed through the option implemented in MS Word 2007 Italian dictionary, as in previous studies on Italian [79]. Gulpease values may range from 0 (*very hard to read*) to 100 (*very easy to read*). The scale of values is further divided into five sub-ranges whose thresholds change according to subjects’ education level.

As regards variables assessed behaviorally, texts including literary metaphors were assigned to four different lists in pseudo-randomized order using a Latin-Square design. The same procedure was applied to the literal counterparts. Each list, including an equal number of passages from poems and novels, was assigned to a different group of 15 subjects who were asked to perform one or two of the tasks, following the same design of Experiment 1. While assessing variables on a scale, subjects were asked to read the whole text but to express their judgments only on the target expression. Each session lasted approximately one hour. Other procedural details were identical to those described for Experiment 1.

### Results

#### Descriptive statistics

The summary descriptive statistics are shown in Table 4. The mean readability index of the whole set of texts was 61. This value falls in the range of easy texts for undergraduates according to the Gulpease scale. The values went from the text including *siepe di scrupoli/hedge of scruples* (Gulpease = 42, easy for undergraduates) to the text including *nidi d’illusione/nests of illusion* (Gulpease = 95, very easy for undergraduates).

**Table 4:** Descriptive statistics of Experiment 2 (in context presentation).

Variable	Min	Max	M	SD	Mdn	α	Skew	95% CI
Gulpease index	42	95	60.82	12.87			1.28	3.19
Cloze probability	0	0.53	0.07	0.13	0		2.17	0.03
Familiarity	1.27	3.40	1.93	0.99	2	0.76	0.92	0.06
Concreteness	1.00	2.87	1.80	0.96	2	0.63	0.45	0.06
Difficulty	1.27	4.20	2.62	1.09	3	0.88	−0.11	0.07
Meaningfulness	1.60	4.47	3.21	1.23	3	0.85	0.10	0.08

Gulpease readability index, Cloze probability, Familiarity, Concreteness, Difficulty, and Meaningfulness of 65 literary metaphors presented in context. Minimum and maximum values, means, standard deviations, medians, α values, skewness values, and 95% confidence intervals are provided.

Predictability was calculated as in Experiment 1. The mean value of the 65 literary metaphors embedded in context was 7%. In most cases, cloze probability was zero. The highest value was recorded for *respiro del mare*/*breath of the sea*, which was completed with the target word 53% of the times (N = 15).

As regards the variables assessed on a scale, data showed a good discriminatory validity since all scale points were used. Interrater agreement within each task was evaluated before further analysis. Cronbach’s alpha was 0.76 for familiarity, 0.63 for concreteness, 0.88 for difficulty, and 0.85 for meaningfulness (N = 15). Thus, values were satisfactory for all tests.

As in Experiment 1, also when embedded in context the overall set of literary metaphors were rated as unfamiliar (M = 1.93; Mdn = 2; 95% CI [1.87, 1.99]). Values ranged from *brivido di ferro*/*shiver of iron*, which reported the lowest familiarity value (M = 1.27; Mdn = 1; 95% CI [1.02, 1.52]), to *velo d’ombra*/*veil of shadow*, which reported the highest value (M = 3.40; Mdn = 3; 95% CI [2.62, 4.18]).

Concreteness ratings of the overall set of items proved skewed towards low values (M = 1.80; Mdn = 2; 95% CI [1.74, 1.86]). The lowest value was reported for *ombre di pace*/*shadows of peace* (M = 1; Mdn = 1; 95% CI [1,1]) and the highest for *velo d’ombra/veil of shadow* (M = 2.87; Mdn = 3; 95% CI [2.09, 3.65]).

The set of literary metaphors in context also reported medium difficulty scores (M = 2.62; Mdn = 3; 95% CI [2.55, 2.69]). Values ranged from *fiamma dell’idea*/*flame of the idea*, which was rated as the least difficult (M = 1.27; Mdn = 1; 95% CI [0.83, 1.71]), to *scintilla di senape/spark of mustard*, which was rated as the most difficult (M = 4.20; Mdn = 4; 95% CI [3.83, 4.57]).

Finally, literary metaphors obtained overall medium-high ratings for meaningfulness (M = 3.21; Mdn = 3; 95% CI [3.13, 3.29]). Values ranged from *anello delle labbra/ring of the lips*, which was assessed as not very meaningful (M = 1.60; Mdn = 2; 95% CI [1.25, 1.95]), to *siepe di scrupoli/hedge of scruples*, which was considered very meaningful (M = 4.47; Mdn = 5; 95% CI [3.85, 5.09]).

#### Relations among variables

As in Experiment 1, Spearman’s correlation coefficients were calculated. The matrix is shown in Table 5. As concerns variables measured objectively, unlike Experiment 1, for literary metaphors in context no significant correlations between Google log-transformed frequency and other variables emerged. Gulpease scores showed only a weak inverse correlation with familiarity (r_s_ (63) = −0.26, p<0.05).

**Table 5:** Spearman’s correlation coefficients in Experiment 2 (in context presentation).

Variable	1	2	3	4	5	6	7
**1. Gulpease index**	–	−0.15	−0.09	−0.26*	−0.20	0.07	−0.05
**2. Phrase frequency** a		–	0.20	0.15	.02	0.02	−0.04
**3. Cloze probability**			–	0.25*	0.28*	−0.15	0.10
**4. Familiarity**				–	0.63**	−0.40**	0.08
**5. Concreteness**					–	−0.40**	0.15
**6. Difficulty**						–	−0.66**
**7. Meaningfulness**							–

Familiarity-concreteness and difficulty-meaningfulness were within subjects correlations.

a Values were log_10_ transformed.

* p<0.05; ** p<0.01.

Variables assessed behaviorally showed several significant correlations. The strongest correlation was between meaningfulness and difficulty (r_s_ (63) = −0.66, p<0.01), suggesting that less meaningful literary metaphors in context were rated also as more difficult to understand. This correlation accounted for the 44% of variance. The second strongest correlation was between concreteness and familiarity (r_s_ (63) = 0.63, p<0.01) which accounted for the 39% of variance, that is concrete items were perceived as more familiar. Difficulty correlated inversely with both familiarity and concreteness (in both cases r_s_ (63) = 0.40, p_s_<0.01); thus, also in context, less concrete and more familiar literary metaphors were judged as more difficult to understand. Each correlation accounted for 16% of variance. Finally, cloze probability showed weak but significant correlations with concreteness (r_s_ (63) = 0.28, p<0.05) and familiarity (r_s_ (63) = 0.25, p<0.05), capturing 6% and 8% of variance respectively.

Note that, contrary to Experiment 1, here meaningfulness did not correlate with familiarity. This may suggest that more familiar items were rated as more meaningful only in absence of a proper context, while the presence of a supportive context caused the variables to act separately. Therefore, even unfamiliar literary metaphors may result interpretable when embedded in context. This pattern is visible, for example, in the item *lama di buio/blade of dark*, which reported low mean scores in familiarity both when presented out of and in context (M = 1.47, and M = 1.93 respectively), but higher meaningfulness scores when presented in context (M = 3.67) than when presented out of context (M = 2.20).

### Interim discussion

The data obtained for the in context presentation showed that (a) literary metaphors were judged as unpredictable, unfamiliar, abstract, difficult to understand yet meaningful; (b) phrase frequency did not interact with other variables; (c) text readability was only weakly related to familiarity; (d) the assessed variables correlated significantly, although no clusters of more than two intercorrelated variables were visible. As in Experiment 1, although the data were obtained from a limited number of participants, the narrow confidence intervals seem to confirm the precision of our estimates and the values of Cronbach’s alpha confirmed the internal consistency of data.

The pattern of the results showed some differences compared to Experiment 1, especially for the absence of a strong correlation cluster. Moreover, mean values of familiarity, concreteness and difficulty, turned out to be lower on average, while cloze probability and meaningfulness reported higher values. In order to better explore these differences, a combined analysis of the two experiments was performed.

## Combined Analysis of Experiment 1 and Experiment 2

A combined analysis of the 65 items included both in Experiment 1 and 2 was carried out on the 65 items included both in Experiment 1 and in Experiment 2. Descriptive scores are displayed in Table 6. The average values of the 65 items from Experiment 1 included in the combined analysis were comparable to the average values of the complete set employed in Experiment 1 (see Table 2).

**Table 6:** Descriptive statistics of items included both in Experiment 1 and Experiment 2.

Variable	Experiment 1 (65 items)		Experiment 2	
	M (SD)	Mdn	M (SD)	Mdn
Cloze probability	0.01 (0.05)	0	0.07 (0.13)	0
Familiarity	2.24 (1.27)	2	1.93 (0.99)	2
Concreteness	2.12 (1.16)	2	1.80 (0.96)	2
Difficulty	3.01(1.15)	3	2.62 (1.09)	3
Meaningfulness	2.62 (1.10)	3	3.21 (1.23)	3

Mean (standard deviations in brackets) and median values for the 65 item subset of Experiment 1, and the 65 items of Experiment 2.

In the combined analysis, Experiment (i.e., absence or presence of the context) was used as a between-subject factor. We compared the scores reported in the out of context *versus* in context condition using the Friedman test for ordinal data and calculated a Spearman rank correlation matrix in order to highlight common patterns.

Friedman tests showed that familiarity (χ^2^ (1, N = 65) = 9.78), concreteness (χ^2^ (1, N = 65) = 23.06), and difficulty rankings (χ^2^ (1, N = 65) = 19.70) were significantly higher for literary metaphors out of context (p_s_<0.05). On the contrary, cloze probability (χ^2^ (1, N = 65) = 13.50) and meaningfulness (χ^2^ (1, N = 65) = 17.79) reported higher values when literary metaphors were presented in context (p_s_<0.001). Note that cloze probability was very low in both cases (M≤7%). Moreover, familiarity and concreteness scores of literary metaphors in and out of context were strongly correlated (respectively r_s_ (63) = 0.67, r_s_ (63) = 0.63; p_s_<0.01), difficulty scores were poorly correlated (r_s_ (63) = 0.27; p<0.05), while meaningfulness ratings were not significantly correlated (p = 0.9).

## General Discussion

In this study we collected word and phrase frequency, readability, cloze probability, familiarity, concreteness, difficulty, and meaningfulness scores for a set of 115 literary metaphors in isolation and for a subset of 65 literary metaphors embedded in their original context. This dataset is the first available repertoire that includes literary metaphors and at the same time takes into account also the context in which the metaphorical expressions originally appear. To the best of our knowledge, this is also the first literary metaphor dataset available for Italian. Due to their regular structure, the materials can be easily sampled and matched with literal or anomalous counterparts to fit the requirement of different experimental designs. Moreover, the materials can be employed in studies comparing literary and non-literary metaphors, as the main psycholinguistic variables employed in studies on everyday figurative language are taken into account, offering a reasonable range of values to allow for independent manipulations. The set can also be useful for studies expanding to context and natural reading of poems and novels.

Although the main contribution of our study relies in the dataset and the descriptive scores, in what follows we would like to point to a number of interesting elements that emerged from the analysis of the data and deserve further investigation in future studies. First, the average scores obtained by the dataset on all dimensions are around medium-low values, and a cluster of properties emerged when literary metaphors were presented as isolated expressions. Comparable results were observed in previous studies on literary metaphors, pointing in the direction of a ‘monster factor’ encompassing different correlated properties [41]. To this respect, literary metaphor does not seem to behave differently from novel everyday metaphors, which too reported medium-low variables in several highly correlated dimensions [39].

The most notable results concern the comparison between in and out of context presentation in the combined analysis of Experiment 1 and 2. The presence of context determined a variation in the ratings on all variables and their correlations. Here special features of literary metaphor seem to emerge, and considerations coming from pragmatics and cognitive poetics might help interpreting them. Based on previous evidence on the role of context in everyday metaphor and language processing in general, one would assume that the textual dimension, i.e., the information provided in the text, has an effect on the psycholinguistic features of the metaphorical expressions, and, specifically, a facilitating role [67], e.g., enhancing predictability, familiarity and meaningfulness, while reducing difficulty. Our results do not entirely support this view. Starting with cloze probability, as expected the values were very low in both conditions, but surprisingly literary metaphorical items proved only slightly more predictable in context than out of context. Moreover, context seems to reduce rather than increase familiarity: literary metaphors were perceived as less familiar when presented in their context than when presented as isolated phrases. These findings mark important differences with respect to the literature on everyday metaphor. In the absence of context, subjects processed the literary metaphorical phrases without interpretational cues. Subjects’ judgments are likely to reflect lexical-pragmatic processes (i.e., adjustments of word meanings to make sense of the expression [80]) that are nevertheless very limited in inferring the intended message. Conversely, in the presence of context, judgments on the phrases are likely to reflect a mechanism that operates more globally [81], as context leads to the construction of situation models and wider interpretative scenarios [82]. Yet the literariness of the contexts employed here might induce global processes that differ from the elaboration of non-literary contexts. While the context of everyday discourse and conversation facilitates comprehension, literary texts seem to promote mechanisms that make the metaphors more open to different interpretations in different scenarios, rather than more familiar. This seems to combine well with the distinctive feature of literary metaphor emphasized in pragmatics, namely the open-endedness of the interpretative activity [24], and also with the narratological approach to metaphor [26].

Another important aspect to underline with respect to predictability and familiarity is related to previous exposure. Although not systematically investigated here, it is possible that the subjects had encountered the texts and the metaphors before, for instance in school. While repeated exposure decreases the familiarity of everyday conventional metaphors (as shown for instance through repetition-suppression paradigms [83]), this does not seem to hold for novel metaphors, and neither for literary metaphors. A remarkable property of literary metaphor is indeed that they do not lose their force after repeated exposure [26]. The array of possible interpretations generated while reading literary metaphors in their original contexts might thus hamper familiarization, making the metaphorical expressions permanently novel and unfamiliar, as a sort of meaning structure constantly open to all possible interpretations driven by the text.

Also concreteness reported lower scores when metaphorical phrases were embedded in context than when presented in isolation. Again, this finding might be motivated in relation to lexical-pragmatic elaboration as opposed to global elaboration induced by literary context. The latter stimulates a deeper and more contextualized interpretation as compared to the former, possibly evoking new interpretative scenarios that potentiate abstract aspects of the metaphor, for instance its emotional engagement [84]. Concreteness at the word and phrase level is likely to be overcome by processes that operate more globally within the text through abstraction, reflecting in lower concreteness judgments on the expression. This is visible, for instance, in predicate metaphors (e.g., *The car flew through the intersection*), where the concrete attributes of verb meaning are overcome by conceptual, abstract attributes [85], and it might extends to other types of metaphors, especially in literary context.

A different trend was observed for difficulty, where expectations of the facilitatory role of context were confirmed: scores were lower (i.e., easier interpretation) when metaphors where presented in the original texts. Furthermore, context enhanced meaningfulness scores, thus making the expressions more interpretable. This is in harmony with previous findings reporting higher meaningfulness judgments for metaphorical items presented as written by poets [31]: literary context increases the recognition of the author’s communicative intentions and aesthetic goals, which enhances interpretability in the subjects’ perception. Taken together, these results suggest that literary context promotes the intensity with which readers search for interpretations and the ease of the process at the same time.

Furthermore, the correlation matrices in the absence and in the presence of context suggest a number of remarks. First, phrase frequency interacted with other variables only when items were presented in isolation, but not in the contextualized presentation. This indicates that, in context, frequency no longer affects the other features of the expression, being overcome by more global properties of the text. Second, when literary context was provided, correlations among variables proved less visible. Notably, meaningfulness was no longer related to familiarity but only to difficulty. This suggests that meaningfulness and familiarity are likely to have a common pattern (more familiar is also more meaningful and vice versa) only when subjects have no textual interpretative cues, and, moreover, that also unfamiliar metaphor may become interpretable when a rich context is provided. Again, this might be due to greater identification of authorial intention in the context condition, through which the particular representation of the world condensed by the author in the metaphor is restored in the reader.

## Conclusions

Overall, our findings provide initial evidence on the phenomenon of literary metaphor in and out of context, by showing that its complexity is highly entrenched with context, in a way that exhibits important differences from everyday metaphors. As pointed out in theoretical studies, literary metaphors appear as overt incongruities that are validated within the interpretation of the text [26], which, in turn, might contribute to evoke the wide range of weakly implicated elements that form the poetic halo [24]. Additionally, the text might promote sensibility for literary metaphors with a higher involvement of cognitive processes on the part of the reader and even a higher attempt to figure out a meaning while recognizing authorial intention. All these aspects impact on traditional measures of familiarity, meaningfulness, difficulty etc. To our knowledge, no previous study reported similar results, and we hope that these findings will be further investigated in future studies exploring the closer relation between figurative language and the text. A crucial aspect to include in future research is aesthetic appreciation, which only recently has started to be considered as one of the variable to assess [86]. Spontaneous aesthetic evaluation takes place during reading, even if not required by the task [87], and this can become extremely pervasive in reading literary text, possibly reflecting a combination of elements that need to be unpacked.

While pinning down the crucial role of context in modulating the impact of the psycholinguistic variables, our results do not offer much evidence in terms of processing mechanisms for literary metaphor, as they do not directly target online processing. However, one might speculate that the traditional dichotomy between indirect and direct accounts possibly fade for metaphors in literary texts, as the very notion of context needs to be rephrased, and cannot be simply considered as an element licensing the figurative expression. Interestingly, in literary texts metaphors frequently extend and occur as chains, and in these cases it is possible that the literal meaning is maintained and meta-represented “as if” the world corresponds to the literal language, in a more reflective and imaginative interpretative mode [12].

We may conclude that literary metaphors turned out to be a very complex phenomenon whose comprehension is influenced and mediated by a cluster of psycholinguistic variables, among which familiarity, difficulty, and meaningfulness emerged. In a more ecologically valid perspective, the literary text as a whole becomes the major element in patterning the psycholinguistic properties of the metaphorical expressions, especially promoting meaningfulness independently of familiarity. We believe that an “experimental turn” in the study of literary metaphor coupled with a “literary turn” in the study of metaphor and context could be key for understanding the distinctive features and possibly the origin of the beauty and emotional impact of certain linguistic expressions.

## Supporting Information

File S1 **Dataset of literary metaphors from the Italian literature.** Table S1, Dataset and descriptive statistics of the literary metaphors assessed in Experiment 1 (out of context presentation) and Experiment 2 (in context presentation). The columns report the following information: metaphorical expression; English word-by-word translation; source; author; descriptive statistics for Experiment 1 and for experiment 2. For Experiment 1, the following scores are reported: FREQ1_value  =  Frequency of the first content word; FREQ1_log  =  Log-transformed frequency of the first content word; FREQ2_value  =  Frequency of the second content word; FREQ2_log  =  Log-transformed frequency of the second content word; GOO_FREQ_log  =  Log-transformed Google-generated frequency counts; CLPR_OUT  =  Cloze probability (out of context); FAM_OUT(M)  =  Familiarity Mean (out of context); FAM_OUT (SD)  =  Familiarity Standard Deviation (out of context); FAM_OUT(Mdn)  =  Familiarity Median (out of context); CONC_OUT(M)  =  Concreteness Mean (out of context); CONC_OUT(SD)  =  Concreteness Standard Deviation (out of context); CONC_OUT(Mdn)  =  Concreteness Median (out of context); DIFF_OUT(M)  =  Difficulty Mean (out of context); DIFF_OUT(SD)  =  Difficulty Standard Deviation (out of context); DIFF_OUT(Mdn)  =  Difficulty Median (out of context); MEA_OUT(M)  =  Meaningfulness Mean (out of context); MEA_OUT(SD)  =  Meaningfulness Standard Deviation (out of context); MEA_OUT(Mdn)  = Meaningfulness Median (out of context). For Experiment 2, the following scores are reported: GULPE  =  Gulpease readability index; CLPR_IN  =  Cloze probability (in context); FAM_IN(M)  =  Familiarity Mean (in context); FAM_IN(SD)  =  Familiarity Standard Deviation (in context); FAM_IN (Mdn)  =  Familiar Median (in context); CONC_IN(M)  =  Concreteness Mean (in context); CONC_IN(SD)  =  Concreteness Standard Deviation (in context); CONC_IN(Mdn)  =  Concreteness Median (in context); DIFF_IN(M)  =  Difficulty Mean (in context); DIFF_IN(SD)  =  Difficulty Standard Deviation (in context); DIFF_IN(Mdn)  =  Difficulty Median (in context); MEA_IN(M)  =  Meaningfulness Mean (in context); MEA_IN(SD)  =  Meaningfulness Standard Deviation (in context); MEA_IN(Mdn) Meaningfulness Median (in context). Empty cells refer to items not included in Experiment 2. Table S2, Dataset of the literary metaphors used in Experiment 2, including introductory sentence, context, source and author.
(XLSX)
